# Legal Framework for Biosphere Reserves as Learning Sites for Sustainable Development: A Comparative Analysis of Ukraine and Sweden

**DOI:** 10.1007/s13280-012-0373-3

**Published:** 2013-03-10

**Authors:** Marine Elbakidze, Thomas Hahn, Volker Mauerhofer, Per Angelstam, Robert Axelsson

**Affiliations:** 1 Faculty of Forest Sciences, School for Forest Management, Swedish University of Agricultural Sciences, PO Box 43, 730 91 Skinnskatteberg, Sweden; 2 Stockholm Resilience Centre, Stockholm University, 106 91 Stockholm, Sweden; 3 6F International Organizations Center Pacifico-Yokohama, United Nations University Institute of Advanced Studies (UNU-IAS), 1-1-1 Minato Mirai, Nishi-ku, Yokohama, 220-8502 Japan; 4 Faculty of Forest Sciences, School for Forest Management, Swedish University of Agricultural Sciences, PO Box 43, 739 21 Skinnskatteberg, Sweden

**Keywords:** Conservation, Development, Informal institutions, Formal institution, Adaptive governance

## Abstract

The Biosphere Reserve (BR) concept aims at encouraging sustainable development (SD) towards sustainability on the ground by promoting three core functions: conservation, development, and logistic support. Sweden and Ukraine exemplify the diverse governance contexts that BRs need to cope with. We assessed how the BR concept and its core functions are captured in national legislations. The results show that the core functions are in different ways reflected in legal documents in both countries. While in Ukraine the BR concept is incorporated into legislation, in Sweden the concept is used as a soft law. In Ukraine managers desired stronger legal enforcement, while in Sweden managers avoided emphasis on legislation when collaborating with local stakeholders. Hence, BR implementation have adapted to different political cultures by development of diverse approaches. We conclude that a stronger legal support might not be needed for BRs, rather SD needs to be recognized as an integrated place-based process at multiple levels.

**Electronic supplementary material:**

The online version of this article (doi:10.1007/s13280-012-0373-3) contains supplementary material, which is available to authorized users.

## Introduction

The Biosphere Reserve (BR) concept was introduced by United Nations’ Educational, Scientific and Cultural Organization (UNESCO) and its Man and the Biosphere (MAB) program in 1974 with two primary goals: conservation and ecological research (UNESCO 1974; Bonheur and Lane 2002; Price 2002). In response to the proliferation of international policies promoting conservation of biodiversity in combination with sustainable use and fair sharing of benefits from utilization of natural resources (CBD 1992), the BR concept has been expanded to also serve as testing grounds for new approaches to sustainable development (SD) as highlighted in the Seville Strategy (UNESCO 1995). The Madrid Action Plan adopted in 2008 further elevated BRs as principal internationally designated areas and ‘learning sites for SD’ (UNESCO 2008). BRs should thus be used by ‘policy professionals, decision-makers, research and scientific communities, management practitioners and stakeholder communities to work together to translate global principles of SD into locally relevant praxis’ (UNESCO 2008). The Rio+20 summit highlighted that BRs should ‘contribute to the transition to green economies by experimenting with green development options including sustainable tourism and training for green jobs’ (UNESCO 2012). It is emphasized that both new and indigenous knowledge should be recognized as input to the SD process. Thus, over the last two decades BRs have changed from being primarily protected areas (PAs) to ‘much more than just protected areas’ (UNESCO 1995). There are currently (October 2012) 610 BRs in 117 countries (UNESCO 2012). The number of BRs is steadily increasing as many countries seek opportunities to promote SD as a societal process and sustainability as the outcome (e.g., Schliep and Stoll-Kleemann 2010; Axelsson et al. 2011).

According to the current definition, each BR is intended to fulfill three core functions: (1) a conservation function to conserve genetic resources, species, ecosystems, habitats, and landscapes; (2) a development function to foster sustainable economic and human development; (3) a logistic support function, to support research, monitoring, education, training, establishment of demonstration sites, and to promote environmental awareness related to local, national and global issues of conservation and SD (UNESCO 1995).

The core functions of BRs should be spatially articulated through area zonation. Formally, each BR should contain three defined management zones. The first is the core area with legally PAs, which may only be entered for purpose of research and monitoring. The second is the buffer zone that is supposed to surround the core area, and is used for low impact tourism, forestry, and agriculture in line with overall conservation objectives. The third is a flexible transition area with a variety of different land use activities, where model projects for sustainable economic development are supposed to be implemented (UNESCO 1995).

BRs can also be seen as multilevel informal institutions (Hahn et al. 2006) because their management plans are not necessarily legally binding. The question of giving BRs legal recognition in national legislation has been a recurring subject of discussions within the MAB Program and regional BR networks. During these discussions, difficulties in management of the transition zone, establishment of dedicated authorities for BRs, and creation of a framework for cooperation among stakeholders were the main challenges (Bonnin and Jardin 2009). The Madrid Action Plan recommended member states of MAB UNESCO Program that ‘Biosphere Reserves receive a reinforced legal recognition, and that Member States are encouraged to include BRs in their legislation’ (Target 11, action 11.1) (UNESCO 2008).

Assessing the legal recognition of BRs as learning sites for SD requires definitions of SD. There is a wide consensus that SD is a continuous process (Baker 2006) and that three main dimensions (environmental, economic, and socio-cultural) should be achieved (WCED 1987). However, there are different opinions about the relationship among the different sustainability dimensions (e.g., Mauerhofer 2008; Blowers et al. 2012). In this paper SD is understood as the societal process of steering towards collective ecological, economic, and socio-cultural goals as envisioned in national and international policies by multiple actors and stakeholders with different power at multiple levels of decision-making (Baker 2006; Strange and Bayley 2008; Axelsson et al. 2011).

The purpose of this paper is to compare how BRs and their core functions as defined by UNESCO are captured and hence supported by national legislation in two countries with different governance systems and political cultures (sensu Katchanovski 2006). We also discuss the normative question of whether the performance of BRs would benefit from a stronger legal recognition and if so, whether a separate law for each BR is preferable. Ukraine and Sweden were used as case studies in our comparative analysis. These two countries represent different parts of the important gradients of landscape history and political culture across Europe (see Angelstam et al. 2013).

## Methodology

In terms of legislation, states form constitutional units, and are thus appropriate units for studies of comparative politics (Landman 2003). For this comparative analysis we employed a set of methods. Viewing each country as a case study (*n* = 2), we treat the BRs in each country’s forest and woodland ecoregions (*n* = 5 + 5) as replicates. First we used the Nomination Forms of each BR to assess which legal documents they refer to. Then we assessed these legal documents quantitatively and qualitatively. Finally we interviewed managers from each BR to understand what different legal documents meant for BR management.

### Countries as Case Studies and BRs as Data Source Replicates

#### Ukraine

The MAB Ukraine National Committee was created in 1973, only 2 years after the MAB program was launched by UNESCO. The first BR was designated in 1984 with the main goal of nature protection. There are currently eight BRs in the country, including both old (UNESCO 1974) and new (UNESCO 2008) generations of BRs. The total area of BRs in Ukraine is about 400 000 hectares. There are plans to establish three new trans-boundary BRs, two along the European Union’s eastern border, and one at the border with the Russian Federation.

A National Committee on SD was established in 2009 with a primary goal to evaluate the implementation of national programs considering economic, ecological, and socio-cultural conditions. It is an advisory body under the Cabinet of Ministers of Ukraine. At the same time, Ukraine faces a number of challenges in realizing SD as a process, including a high level of corruption, a poorly developed democracy and inadequacy of institutions (Katchanovski 2006; Gorobets 2008).

Five BRs (out of 8 in the country) were chosen as replicates for the Ukrainian case study (Electronic Supplementary Material, Table S1). The Carpathian BR belongs to the first generation of BRs based on the legal status before 1995 (i.e., before the Seville Strategy) with the main focus on nature conservation. The other four BRs belongs to the second generation of BRs after 1995, and thus aiming to be learning sites for SD and nature conservation. All BRs in Ukraine have their core areas and buffer zones free from permanent inhabitants. At the same time the transition zones have diverse type of land use activities and are home to many people.

#### Sweden

In Sweden, work to promote environmental sustainability started early. The Stockholm conference in 1972 is considered as a starting point of the SD concept. The interest for BR development is, however, comparatively recent (Hahn 2011). The first BR Abisko appeared in 1986, but was later excluded after an initiative by the Swedish government because it did not meet the requirements of the current MAB statuary framework for BRs (Schultz and Lundholm 2010). The new generation of BRs in Sweden began to appear in 2005, and currently there are five BRs with the total area of around one million hectares. All these five were selected as replicates (Electronic Supplementary Material, Table S1). Diverse land use activities are conducted in all management zones, including the core areas where certain types of land use are important for nature conservation.

The Swedish National MAB Committee is hosted by the Swedish Environmental Protection Agency. The MAB Committee is the decision-making and funding committee for MAB activities in the country. There is no special law for BRs, but Sweden aims to reflect international agreements in its national law. Hence, all issues related to management and governance of BRs have to be solved based on more general national legislation and policies.

The selected BRs in Sweden are appropriate for a comparative analysis as they are comparable with the selected BRs in Ukraine when it comes to location in forest ecoregions, a diversity of land use activities associated with the forest landscapes, and thus by laws regulating the use of natural resources in both countries.

## Methods

### Identification of Relevant Legislation

Institutions are the rules and norms of action in society. While formal institutions refer to laws and regulations that are enforced by a third party, informal institutions are the social norms and conventions that are not externally enforced (Bromley 1991). The official Nomination Forms during the designation procedure for submission to National Committees in Ukraine and Sweden to become a BR MAB were prepared by each of the 10 case study sites. These Nomination Forms were used to identify the formal institutions (i.e., legal documents such as laws and policies) relevant to the three core functions. The main source of information in each Nomination Form was chapter 17 ‘Institutional aspects’. Additionally, chapters 13, 14, and 15 describing the core functions of BR were read to check if all laws and policies were reflected in chapter 17. The legislation that deals with the core functions of BRs in both countries were analyzed using quantitative and qualitative methods.

### Quantitative Analysis

Inspired by the quantitative part of the ‘Legislation-Check’ approach developed by Mauerhofer (2012), we picked up keywords describing the core functions of BRs from the ‘model law’. This ‘model law’ was proposed by the MAB Program at UNESCO as a blue-print for states wishing to elaborate a specific legal category for BRs (Bonnin and Jardin 2009). There are at least two reasons for choosing the ‘model law’ as a source for keywords. First of all, being based on an analysis of already existing national legislation related to the BR concept in 30 countries (Bonnin and Jardin 2009), this model law is rooted in and captures the main elements of the Seville Strategy, the Statutory Framework of World Network of BRs and the Madrid Action Plan that are the main international policy documents for BRs. Second, the ‘model law’ is officially referenced by UNESCO due to its development as a response to discussions within the MAB Program about the importance of BR recognition in national legislation.

The selection of keywords was organized in two steps. First, we selected words from each article of the ‘model law’. There are 15 Articles that deal with the (a) definition of a BR (Article 1), (b) the designation process (Articles 2–4), (c) objectives (Articles 5–8), (d) territory (Articles 9–11), and (e) integrated management of BRs (Articles 12–15). In total 52 keywords were selected. Second, we grouped these selected keywords according to the core functions of BRs; the keywords that reflect a conservation function (in total 10 words); a development function (31); and a logistic support function (11) of BRs (Electronic Supplementary Material, Table S2).

The occurrence of the selected keywords was used to analyze to what extent national legislation literally captures the intended three core functions of BRs. In many cases the search was done for each separate word when a keyword was represented by a term or if the meaning of a keyword was wide. For example, the keyword ‘public stakeholders’ is very general and therefore we also searched for synonyms such as: ‘governmental organization’, ‘municipality’, ‘government’, etc. Another example, a keyword ‘economic development’ was searched using the separate words ‘economic’ and ‘development’ to see if these words were present in connections with other words in national legislation with the meaning of ‘economic development’.

The keywords were translated to Ukrainian for checking the Ukrainian legislation. As the endings of keywords vary in Ukrainian language, all variations were used to implement an assessment similar to the quantitative part of the ‘Legislation-Check’ (Mauerhofer 2012). For the Swedish legislation the majority of laws were available in English, however, several laws were checked using also keywords in Swedish.

### Qualitative Analysis

The legal documents identified in the nominations were further read to understand how the conservation, development and logistic support functions of BRs were defined in the national legislation in both countries. This step is necessary because SD could be addressed in the legislation even without using the key terms searched for during the quantitative analysis (Mauerhofer 2012).

### Expert Interviews

Additionally, to understand how the national laws and policies were used in management of BRs we conducted expert interviews (sensu Flick 2006) with all main managers (*n* = 10) of the selected 10 BRs and with coordinators of the National MAB program in both countries (*n* = 2). The interviewees represent the total population of BR managers in the replicates. An interview guide was developed that contained questions about the legal framework used by managers to fulfill the core functions of BRs. In Ukraine we also discussed how the legal interpretation of BRs in the Law on Nature Protected Area Fund of Ukraine was used by managers to establish BRs as learning sites for SD. Interviews were done in Ukrainian or in English, lasted 1.5–2 h, were recorded digitally and transcribed.

## Results

### Laws and Policies Referred to in the Nomination Forms

The results from the Nomination Forms suggest that the core functions of BRs are legally supported in both countries although they differed in the number of documents found, the extent of the binding character of these documents and the releasing authority (Table 1). In Ukraine the BR concept is literally incorporated into the Law on Nature Protected Area Fund of Ukraine (1992). Despite its update in 2010, it has not been adapted to the new functions of BRs serving as testing grounds for SD. In this law BRs are presented as a specific type of PA of international importance translated as ‘biosphernyy zapovidnyk’. In direct translation this means ‘biosphere strict protected reserve’. In Ukraine, strict protected reserves are established for nature protection only and all kinds of human use are excluded. Additionally, seven laws and two state programs that were supposed to be used to fulfill the main functions of BRs were indicated in the Nomination Forms of the five selected BRs (Table 1).

**Table 1:** The main legislation used in management of BRs in Ukraine and Sweden

Ukraine		Sweden	
	Year		Year
On Environmental Protection	1991	The Nature Conservation Act	1976
On Nature Protected Area Fund	1992	The Hunting Act	1987
On Fauna	1993	The Heritage Conservation Act	1988
On Flora	1993	The Environment Act	1988
The Forest Code	1995	The Heritage Conservation Ordinance	1988
On the Red Data Book of Ukraine	1998	The Cultural Monument Act	1988
The Water Code	2001	The Forestry Act	1993
The Land Code	2002	The Fisheries Act	1994
The State Program on Development of National Ecological Network of Ukraine in 2000–2015	2000	The Environmental Code	1999
The State Program on Forests of Ukraine in 2002–2015	2002	The Planning and Building Act	1994
		The Railways Act	1995
		The Road Act	
		The Species Protection Ordinance	2007
		Environmental Quality Objectives	1999

The four recent BRs in Ukraine established after 1995 (Electronic Supplementary Material, Table S1), trying to satisfy the requirements of Seville Strategy and the Madrid Action Plan, were established ‘outside’ the Ukrainian legislation. It means that these BRs were approved by UNESCO, but not by the Ministry of Ecology and Natural Resources as required by the national law. They have thus not gained any legal recognition by the Ukrainian government. Therefore, according to Ukrainian law there are presently (2012) four BRs, and according to UNESCO there are eight BRs in the country.

In Sweden, BRs have not been literally incorporated or even defined in any law. In the Nomination Forms of BRs 13 legal acts and the national Environmental Quality Objectives (Table 1) were used. Analyzing the expressions of the interviewees, the BRs concept has been used as a soft law (sensu Kirton and Trebilcock 2004) to promote and test different approaches to SD (e.g., collaboration with local stakeholders) and sustainability (e.g., including active nature conservation measures). The BR itself was re-introduced in Sweden by a local actor who was able to develop this idea on the ground (in Kristianstad) by establishing multilevel collaboration with stakeholders from different societal sectors and, later, to scale up this concept to the national level (Olsson et al. 2004; Hahn 2011). To avoid misunderstanding of the BR concept by local stakeholders, the word ‘reserve’ that may create suspicion about possible restrictions on land use for landowners was replaced with the word ‘area’. Hence, BRs are named ‘Biosphere Areas’ in Swedish. Existing collaboration among stakeholders and leadership by actors were the most important foundations to establish all five BRs.

### Quantitative Analysis of Core Functions of BRs in the National Legislation

**Fig. 1:**
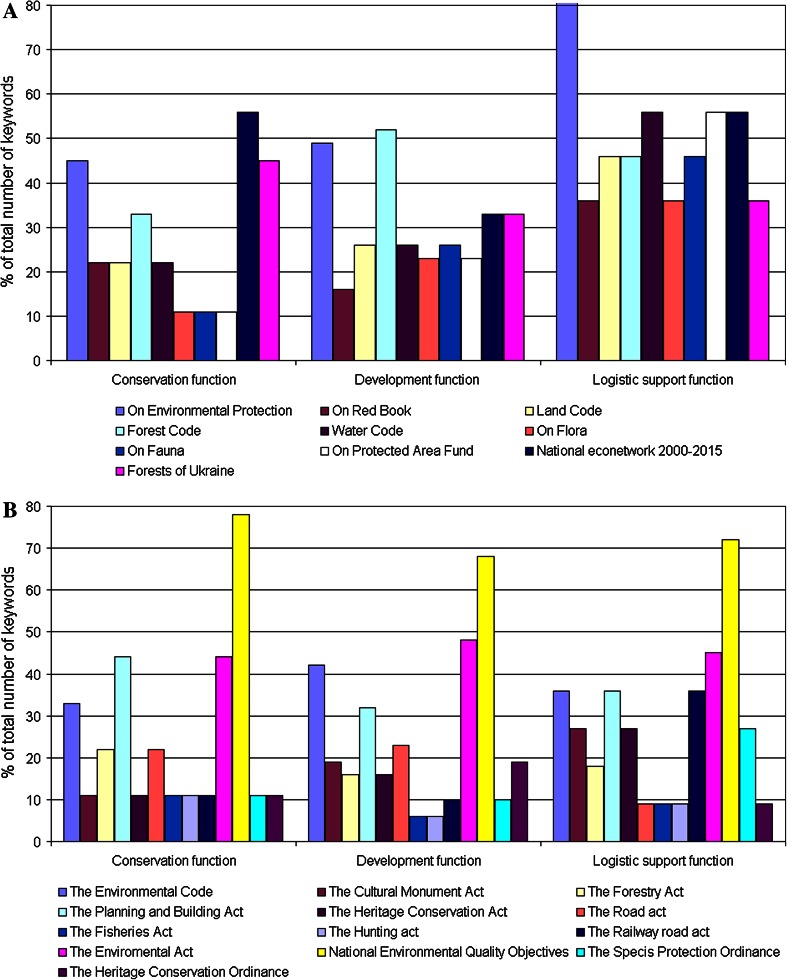
Percentages of verbal keyword reflections for BR core functions in the national legislation (Table S1) in Ukraine (**A**) and Sweden (**B**)

The analysis of the 52 keywords shows that in general the core functions of BRs were verbally captured in the identified legal documents in both Ukraine and Sweden. However, there was no single law that covered all of them (Fig. 1a, b). In the Ukrainian legislation the logistic support function was best captured: 7 of 10 legal documents contained more than 40 % of these keywords. The conservation function was verbally reflected more than 40 % only in two State Programs and in the Law on Environmental Protection; and the reflection of the development function in the Law on Nature Protected Area Fund and the Law on Environment Protection also reached more than 40 % (Fig. 1a).

In the analyzed Swedish legislation the distribution of keywords was uneven. For example, 7 out of 13 legal documents contained approximately 10 % of keywords related to conservation function and nearly 20 % related to development function. However, all three core functions were captured by the Environmental Quality Objectives (about 70 % of keywords), and the Environmental Act (almost 40 %).

The keywords had different frequencies of appearance in the legal documents in both countries (Table 2). In Ukraine seven keywords appeared in all legal documents: ‘conservation/protection’, ‘public stakeholders’, ‘social stakeholders’, ‘coordinate’, ‘local’, ‘national’, and ‘environmental monitoring’. In Sweden only ‘conservation/protection’ appeared in all legal documents. On the other hand, missing words were quite similar in both countries. ‘Adaptive management’, ‘adaptive governance’, ‘integrated governance’ are examples of missing words in all legal documents.

**Table 2:** Appearance of keywords in the analyzed legal documents in Ukraine (UA) and Sweden (SE)

	H	M	L	Missing
A conservation function:				
Conservation/protection	UA, SE			
Biosphere			UA	SE
Ecologically sustainable/sustainable environment			UA, SE	
Biological diversity		UA, SE		
Integrated management		UA	SE	
Natural heritage/asset		SE	UA	
Long-term protection			SE	UA
Ecological functions			UA, SE	
Ecological connectivity		UA		SE
A development function:				
Economic development		UA	SE	
Human development			UA	SE
Socio-culturally sustainable		UA	SE	
Sustainable development		UA	SE	
Future generations			UA, SE	
Cultural heritage	SE	UA		
Multiple-use				UA, SE
Sustainable use		UA	SE	
Ecologically healthy		UA	SE	
Economically viable			UA, SE	
All concerned parties		UA, SE		
Private stakeholders	UA, SE			
Public stakeholders	UA, SE			
Social stakeholders	UA, SE			
Natural products			UA	SE
Ecosystem services			UA, SE	
Adaptive management			SE	UA
Integrated management		UA	SE	
Adaptive governance				UA, SE
Appropriate technologies		SE	UA	
Traditional knowledge			UA, SE	
Local communities			UA, SE	
Participation	UA	SE		
Integrated management policy				UA, SE
Integrated governance				UA, SE
Coordinate	UA	SE		
Integrate			SE	UA
Consultation	SE		UA	
Interaction				UA, SE
National and regional development policy			SE	UA
Land development		SE	UA	
A logistic support function				
Exchange of experience			UA	SE
Environmental education		UA	SE	
Local	UA, SE			
Regional	UA, SE			
National	UA, SE			
Global/international	UA, SE			
Awareness			UA, SE	
Interdisciplinary			UA	SE
Innovation			UA	SE
Environmental monitoring	UA		SE	
National communication			SE	UA

In the columns indicated as ‘H’, ‘M’, ‘L’, and ‘Missing’ show an appearance of a certain keyword in a number of laws: H (high) means that a certain keyword appeared in more than 60 % of analyzed legal documents; M (moderate) from 30 to 60 %; L (low) in less than in 30 %; and Missing means it was absent in all analyzed legal documents

The proportion of missing keywords was, for Ukraine and Sweden, respectively, highest for the development function (26 and 23 %, respectively) followed by the logistic support function (9 and 27 %, respectively) and conservation function (10 and 20 %, respectively). This suggests that the Ukrainian legislation incorporates the core functions to a higher extent than the Swedish legislation except for the development function.

### Qualitative Analysis of National Legislation Related to Core Functions of BRs

#### Conservation Function

In Ukraine the conservation focus in the national legislation covers a wide range of objectives such as protection and conservation of genetic resources, species (e.g., species diversity including habitats, integrity of natural communities of species, population and genetic diversity, etc.), and ecosystems and landscapes (e.g., representative and unique natural complexes; landscape diversity; green space of urban areas, etc.). For example, Article 3 in the Law on Environmental Protection states that ‘conservation of spatial and species diversity and integrity of natural objects and complexes’ is one of the basic principles of environmental protection.

In Sweden the objectives for conservation, protection or preservation are described in more general terms such as protection and preservation of natural and cultural environments; biodiversity (e.g., natural habitats and of wild species); unspoiled nature, or area of particular importance for the protection of certain species of birds. At the same time, the Environmental Quality Objectives state that ‘Biological diversity must be preserved and used sustainably for the benefit of present and future generations. Species habitats and ecosystems and their functions and processes must be safeguarded. Species must be able to survive in long-term viable populations with sufficient genetic variation’. In many cases in the Swedish legislation, the keyword ‘protection’ is connected to protection of natural resources, agricultural and forest land, land for outdoor recreation, commercial fishing, reindeer husbandry, and for industrial development. Thus, in many cases protection relates to both biodiversity and objects important for human well-being, primarily human health. The Environmental Code’s Section 1 thus states: ‘Sustainable development will be based on recognition of the fact that nature is worthy of protection and that our right to modify and exploit nature carries with it a responsibility for wise management of natural resources’.

Additionally, in both Ukraine and Sweden much attention was paid to protection of land (land resources, natural ecological values of land), water (hydrological, hydrobiological, and sanitary conditions of rivers), and forests (recreational and protective functions of forests; biotic and other natural diversity of forests).

In the analyzed Ukrainian legislation there were prescriptions to maintain ecological networks in the Forest Code (Articles 14, 19, 20, 46), the Law on Environmental Protection as well as the Law on Red Book. A definition of ecological network is given in the State Program on Development of National Ecological Network of Ukraine in 2000–2015 where the following is stated: ‘Ecological network is an integrated territorial system that includes natural landscapes that are under protection; PAs; areas with health and therapeutic, recreational, water and field protective functions and other objects that are defined according to the Ukrainian legislation and are an integrated part of natural regions, natural corridors and buffer zones’. The Forest Code prescribes that all permanent and temporary forest uses and forest owners have to assist to the development of ecological networks; and representative and unique natural complexes, old-growth forests and habitats of red-listed species of flora and fauna that are a subject of conservation and a part of ecological network should be considered in the forest management plans (Article 46). In the analyzed Swedish legal documents the term ‘ecological connectivity’ is used only in relation to Natura 2000 network in the Environmental Quality Objectives. Issues related to connectivity are instead defined and implemented into tactical spatial planning by county administrative boards (see Angelstam et al. 2011).

There is only one legal document in Ukraine, the State Program on Forest of Ukraine, where the term ‘ecologically sustainable’ appears, but in a very general interpretation. Regarding ‘integrated management’, there is only a general use of the term in the Law on Environmental Protection, Land Code, Water Code, and Law on Fauna. In Sweden, in the Environmental Quality Objectives, 16 such objectives have been defined in order to achieve a desirable environmental state in a specified time period. In the description of each objective and their interim targets there are certain requirements related to ecological sustainability. For example, the 8th Environmental Quality Objective titled ‘Flourishing lakes and streams’ states that ‘Lakes and watercourses must be ecologically sustainable and their variety of habitats must be preserved’. Regarding ‘integrated management’ in the Swedish documents the term ‘integrated approach’ is used instead. The Building and Planning Act and the Environmental Quality Objectives state that sustainable use of land and water ‘call for an integrated approach to the landscape, in which public health, natural and cultural considerations all have a place’.

#### Development Function

In the Ukrainian legal documents, SD is mentioned in the Law on Environmental Protection, the Forest Code of Ukraine and in the National Program ‘On Forests of Ukraine in 2002–2015’. In the Swedish legislation the term SD is used in the Environmental Code, the Environmental Act and the Environmental Quality Objectives. However, although the term is included, there is no clear definition of SD in the analyzed legal documents in both countries. For example, in the preface of the Law on Environmental Protection in Ukraine it is declared that ‘Environmental protection, rational use of natural resources, and ecological safety for human life is an essential condition for sustainable economic and social development of Ukraine’. The focus of Forest Code of Ukraine and the National Program ‘On Forests of Ukraine in 2002–2015’ was to secure the sustainable forest management in order to enforce ecological, social and economic functions of forests. In Sweden, the Environmental Code states the purpose of the Code is ‘to promote sustainable development which will assure a healthy and sound environment for present and future generations’. In order to achieve sustainability the objectives of the Environmental Quality Objectives are defined as ‘to protect human health, to preserve biological diversity, to minimize the utilization of natural resources to ensure sustainable use and to protect the natural and cultural environment’.

There were prescriptions related to sustainable use of natural resources in both countries. However, the Ukrainian acts do not specify what this means and how to do it. The Swedish Environmental Quality Objectives in the chapter ‘A strategy for the management of land, water and the built environment’ declare in order to ensure the sustainable use of natural resources that ‘all sectors and actors have a common responsibility to take the whole landscape into consideration when planning or engaging in resource utilization’. The duties for certain governmental organizations are defined towards coordinating efforts ‘to draw up regional landscape strategies for biological diversity at county level’. Additionally, those organizations ‘should provide guidance on landscape strategy development and planning, in consultation with other relevant central agencies as well as a proposal for implementation at the national level of the European Landscape Convention’. This chapter also states that in order to facilitate practical application of ‘sustainable use of biological diversity and biological resources’, the Government also intends to commission the sectorial agencies responsible for land-based industries to further define and develop the concept.

Economic development was not explicitly presented as a goal in the analyzed legal documents in either of the two countries. Rather, natural resources were considered as a foundation for economic and social development. Human development appears only in the Ukrainian State Program on Development of National Ecological Network; but rather as consequence of—than a goal towards—successful implementation of the Program. It says that ‘Implementation of the Program will ensure conservation and restoration of landscape diversity, and also maintain ecological balance of the territory of Ukraine. Further, also create natural conditions for human’s life and development in ecologically balanced natural environment’.

There were many references to private, public and social stakeholders in all (Ukraine) or most (Sweden) legal documents. The majority of provisions are about rights and responsibilities of private and public stakeholders to fulfill certain actions. In Ukraine all analyzed laws prescribe that private and social (or civic) stakeholders have rights to participate in diverse activities performed by the governmental organizations (public stakeholders) related to protection/conservation, restoration and use of natural resources and PAs; to inspect how different users use natural resources; to conduct civic ecological expertise of natural resources and PAs with announcement of its results; to conduct a civic control on use and protection of natural resources and PAs; to have an access to information about state, use and protective measures related to natural resources; participate in management of PAs, etc. In the Swedish legal documents there are many prescriptions related to protection of private and public interests or ownership rights. Situations with conflicting interests related to use of land and water and biological resources, should be solved through consultations, dialogues or collaboration according to Swedish laws.

#### Logistic Support Function

The Ukrainian legal documents contain provisions on mainly basic and applied disciplinary research related to local and national issues of conservation and use of natural resources. Only in the Law on Environmental Protection Article 3 ‘Basic principles of environmental protection’ lays down that one of the principles such as ‘integration of ecological, economic and social interests of society based on interdisciplinary knowledge of ecological, social, natural and technical sciences’. In Sweden only two analyzed legal acts refer to research in a quite general sense. The Environmental Code tells about marine research, and in the Environmental Quality Objectives it is declared that ‘Continued research in all areas is essential if action on the environment is to move forward and the Environmental Quality Objectives and their interim targets are to be achieved. There is also a need to intensify research efforts in the social sciences into methods and policy instruments to be used in pursuit of the Environmental Quality Objectives’.

Environmental monitoring is a goal and a duty of certain governmental organizations in both countries. The laws contain prescriptions about how environmental monitoring should be organized and conducted. All analyzed Ukrainian laws refer to environmental monitoring in general or to monitoring of certain objects (land, water, fauna, flora, forests and national ecological networks) that are considered as an integral part of environmental monitoring itself. In Sweden environmental monitoring is considered only in the Environmental Quality Objectives which state that ‘Environmental monitoring is an important tool as it provides supporting data for ongoing revision of Environmental Quality Objectives and interim targets, serving as a basis for future action’. Environmental education was considered in legal acts both in Ukraine and Sweden.

### Managers’ Perspectives on Legislation for BRs

In Ukraine all interviewees confirmed that the main law applied in management of BRs was the Law on Nature Protected Area Fund of Ukraine (1992). But all of them pointed out that this law was too narrow to fulfill all core functions of BRs, especially the development function. Trying to solve this inconsistency, the representative of MAB Ukraine proposed two options: (1) to improve the current law; or (2) to develop a separate law for BRs which takes into consideration the model law proposed by MAB UNESCO instead of an existing one. However, there is no policy action in any direction yet.

One interviewee explained the need to apply legislation for implementation of BR’s ideas on the ground:

Local communities, especially new businesses do not follow the legislation and are trying to get only own benefits. This is a reason for conflicts not only with the nature conservation law, but with other laws as well.

As mentioned in several interviews, the management of BRs is done top-down by introducing restrictions of land use activities in the areas. One interviewee commented on this:

It was the state land before 1991. And probably our management is to some extent totalitarian with a government which people do not like. At the same time, we can not change anything only by talking to people. On some stages law is a strong and influential mechanism to move towards sustainability.

The interviewees also mentioned other legal acts that they were able to use in their management, such as the Land Code, the Water Code and the Forest Code. On the question what challenges the BRs’ managers met when implementation BR ideas on the ground, they referred to the absence of funding, and an appropriate legal control on land use activities in the transition zone. Interviews with managers of new generations of BRs suggested that management of BRs did not differ much from management of PAs. These strictly protected reserves and national nature parks served as a “land platform” for establishment of BRs.

In Sweden, according to the interviews, all five BRs emerged from different projects initiated by local actors. Financially, the BRs and their projects were supported by EU, municipal or other funding to monitor, conserve, protect, or restore ecological values of landscapes that were associated with human activities. All interviewees stated that if BRs would have a legal status regulating land use similar to PAs, BRs would be absolutely impossible to establish. Landowners with their strong position in legislation and culture in Sweden are skeptical of top-down decisions influencing their property. One interviewee explained this:

If we had said to local people that we would create a BR and it would mean a stronger legislation we would be forced to forget about the idea of BRs in Sweden at all. We are saying that we are a BR, we have the ordinary legislation and are trying to understand how a BR could be used to conduct our activities in a sustainable way. When we talk to farmers we put legislation aside, we should not be a police. Quite often there is no sharp edge between what is allowed and not allowed to do according to the legislation. We try to find a positive way to start talking to farmers and make them proud of their land.

The interviewees argued that as a soft law the BRs gave more space for new methods and ideas in natural resource management, including ideas for integrated spatial planning compared to existing legislation in Sweden.

‘The fact that a BR in Sweden is not a legal instrument makes it interesting to work with. Our purpose is actually to work not with biodiversity that is extremely high in the core zone, but to work with people for increasing their knowledge, to help them understand why we need to do certain things and explain long term effect of our choices’, commented one interviewee. The European Union’s funding for implementation of international and EU policies such as Landscape Convention, Common Agriculture Policy, Convention on Biological Diversity and rural development was mentioned as important financial mechanisms for development of BRs.

## Discussion

### Meaning of ‘Legal Recognition’ of BRs as Learning Sites for SD

This study suggests that formal institutions are very important for governing BRs; both countries refer to a range of legislation used to support the core functions of BRs. The legal analysis shows that all three core functions of BRs are captured in national legislation in both countries. In Ukraine, the BR concept is incorporated into the Law of Nature Protected Areas Funds since 1992 but this law has not adapted to the evolution of the BR concept to make BRs learning sites for SD. However, this legal ambiguity has not stopped the development of new BRs in Ukraine.

The quantitative analysis of keywords in legal texts showed that, even if only considering a limited number of legal documents in each country, the analyzed legislation verbally reflected almost 80 % of the 52 keywords derived from the ‘model law’. The core functions of BRs were best verbally reflected in the ordinary environmental legislation in both countries, as well as in the governmental programs (in the two State programs in Ukraine and in the Environmental Quality Objectives in Sweden). Thus, the current legislation in both countries might be used to drive the BRs agenda forward.

Based on the qualitative analysis of national legislation related to core functions of BRs, in spite of the differences in governance, we argue that there are opportunities to fulfill all functions in both countries. There are, however, different challenges as well. Regarding the conservation function of BRs, the prescriptions related to conservation of biodiversity in terms of composition, structure, and function of ecosystems exist in the analyzed laws in both countries. The conservation function is spatially connected to all management zones of BRs. However, the majority of species and their habitats that are targets of conservation efforts are located in the core zone of BRs. In all analyzed BRs the core zones consisted of legally recognized PAs: the strict protected reserves and strict protected zones in the national nature parks in Ukraine, and nature reserves, nature conservation areas, Ramsar sites and Natura 2000 in Sweden. The PAs are established according to the national environmental legislation (in both countries) and in parallel to the EU’s Directives (in Sweden). The main difference among the two countries was that in the Ukrainian BRs, protection in the core zone included restrictions to all kinds of human economic activities including permanent residence. By contrast, Swedish BRs are based mainly on encouragement and maintenance of certain types of traditional land uses important for biodiversity of cultural landscapes. Beyond the core zone there are also legal opportunities in both countries to bring ecological considerations to land use activities. This is clearly stated in the environmental as well as forestry, water, and land management related legislation.

Regarding the development function of BRs, there are also opportunities to move the SD agenda forward. At the same time, for example, in Ukraine many laws refer to sustainable use of natural resources and encourage collaboration among private, public and civil stakeholders. It can be used as a good legal foundation to fill these prescriptions with real content through testing different approaches and practices in the BRs. Similarly, in Sweden, the Environmental Quality Objectives could serve as a legal ‘backbone’ for sustainable economic and social development. The landscape strategy development and planning through collaboration with diverse stakeholders that is prescribed as duties for certain governmental organizations by the Environmental Quality Objectives could be used by BRs as an approach and a tool to integrate ecological, economic and socio-cultural dimensions of SD in their areas. Additionally, projects funded by the EU (Life, Leader plus, etc.) are used to encourage SD for economic and social development in the Swedish BRs. Nevertheless, there were no clear definitions of SD in any analyzed legal document in both countries.

The logistic support function of BRs is verbally best reflected among the three core functions in both Ukraine and Sweden. Probably the reason is that it is mainly related to ‘soft’ prescriptions concerning research, education or monitoring. While the legal framework of SD is not well developed in either of the two countries, there is an urgent need to have ‘places and spaces’ where different aspects of SD are tested and implemented, including legislation. For example, even if the Ukrainian legislation mentions ‘transdisciplinary knowledge’ for integration of disciplinary research and practice only once, still it could be used as an argument to develop this important topic further by supporters of SD agenda, including BRs’ managers (Axelsson et al. 2011; Angelstam et al. 2013). At the same time, prescriptions to conduct environmental monitoring are in the majority of analyzed Ukrainian legal documents and in the Environmental Quality Objectives in Sweden. These legal documents could be used as a tool to perform the core functions of BRs by obtaining important data needed for better spatial integrated planning and to navigate BRs’ agenda towards a desirable direction.

Thus, the pool of institutional arrangements in Ukraine ensures that all core functions might be supported. Hence managers have to rely on various legal documents, not only the specific law that was designed to govern BRs. This is supported by interviewed managers in this study, and by another study of the establishment of a BR in Ukraine (Elbakidze et al. 2013). At the same time informal institutions, the norms and expectations of different stakeholders seem to be crucial for the performance of the BRs. The Swedish BR managers also have to rely on various legal documents although they avoid emphasizing legislation while working with local stakeholders. The BR concept is used as a soft law without any explicit reflection in the national legislation but still unintentionally supported by several laws, especially the National Environmental Quality Objectives. Any legal recognition of BRs has been resisted by the Swedish managers. The opposite is true in Ukraine where managers relay on financial support from the governments and legal control mechanism. Nevertheless, a separate law for BRs inspired by the ‘model law’, as suggested by the UNESCO MAB Office, might be a viable option for Ukraine as proposed by the representative of MAB Ukraine, and—at least—a political option for Sweden. Thus, to adapt the governance and management of BRs to the new expectations of becoming learning sites for SD (UNESCO 2012), we suggest that institutional flexibility is important for adaptive governance of BRs.

In our study we analyzed only those legal documents which were listed in the Nomination Forms of BRs selected as case studies. It was done on purpose because it made easier to select legal documents for our analysis. In order to have a full picture of legal opportunities for BRs to fulfill their functions, we would need to consider and analyze all legal documents, including laws, policies and governmental strategies in each country. The simple overview of identified legal documents provided in this paper gives general arguments about opportunities and challenges which BRs might have in fulfillment their functions. We found that existing legal frameworks in both Ukraine and Sweden create opportunities to fulfill the core functions of BRs.

### Legitimization for Biosphere Reserves and Legislation for SD

The interviews with managers of BRs in Ukraine and Sweden showed that they have a similar understanding of the role of laws in their daily activities: laws are tools that restrict or limit land use activities conducted by local stakeholders. However, in Ukraine managers desired to reinforce their power by having a legal mandate whereas in Sweden managers avoided to emphasize legislation when collaborating with local stakeholders.

We suggest that implementation of BR as learning sites for SD requires a change in culture and behavior of BRs’ managers in Ukraine. The managers of BRs belonging to ‘old generation’ (pre-Seville) have a legal power to manage BRs and their activities are funded by the government according to the Law on Nature Protected Area Fund. Working with a new generation of BRs that are established ‘outside’ of the national law, managers might have to understand BRs as a societal process and be able to perform their activities through collaborations with stakeholders from different societal sectors at different levels without governmental funding. In contrast, Swedish BRs have emerged from collaborations among stakeholders facilitated by local actors through significant funding, and the Swedish government even excluded the oldest Abisko BR that was not based on collaboration.

Ukraine is not alone in struggling with the transition from the old BRs to the new. A global survey of 148 BRs revealed that only 79 of them serve as ‘potential learning sites’ (Schultz and Lundholm 2010). The main challenges identified were establishing platforms for mutual and collective learning through face-to-face interactions; coordinating the generation of new social–ecological knowledge, and framing information and education for various stakeholders. Additionally, Schultz et al. (2011) pointed out that adaptive co-management practices in BRs across the world correlated to a higher level of effectiveness in achieving development goals, and this higher effectiveness did not seem to be at the expense of biodiversity conservation. Our results support that the main challenge for ‘modern’ BRs is investments in informal institutions such as trust-building and collaboration to change social norms and increase legitimacy for a more sustainable use of ecosystems.

The current paper does not fully address the effectiveness of the whole framework of binding and non-binding policies affecting the BRs assessed. It tries to bring some new light onto the complex situation that BR managers face within different implementation approaches for BRs as learning sites for SD. A more comprehensive picture of the ecological, social, and economic implications towards sustainability would be provided through the inclusion of further stakeholders (such as scientists, land owners, and NGOs) in particular into the qualitative methodology.

A tentative conclusion is that a stronger legal support might not be needed for BRs, but instead SD needs to be recognized as an integrated place-based process at multiple levels. Legislation could prescribe how, for example, environmental assessment, research funding, and public consultation relate to SD, and their role in the overall framework for implementation (Ross 2008, 2010). BRs as learning sites for SD could be used to test the legal framework for SD, how it works, opportunities and gaps, and how it could be improved to drive SD in a country forward. This is urgently needed in Ukraine where the political system is unstable, which ultimately creates short-term perspective in governmental ‘thinking’. In this case BRs could maintain important landscape values and support social procedures such as consultations and participation important for SD. In Sweden, BRs might support understanding about how to make stakeholders at multiple levels understand ‘what is at stake’ within the framework SD agenda.

The BR concept is one of many international and national concepts aiming at SD towards sustainability. Other concepts include Model Forest, Local Agenda 21, EU Leader, LTSER, Polish Forest Promotional Complexes (Elbakidze et al. 2010; Axelsson et al. 2011; Blicharska et al. 2012). Such concepts need to be widely accepted by stakeholders as legitimate, and this will improve their usability, and ‘to shift our emphasis from managing resources to managing ourselves so that we learn to live as part of nature’ (Wackernagel and Rees 1996).

## Conclusions

This study stresses the need for differentiated and adapted solutions to implement the BR concept on the ground in different societal contexts. BR core functions were supported by legal documents in both countries. However, the ultimate purpose of BRs is to promote SD and the legal support for this is ambiguous in both countries. BRs as learning sites may develop best practices for trade-offs among three pillars of sustainability by means of differentiated binding and non-binding policy mixes, addressing the relative importance of its three core functions within the BRs’ zonation at different spatial scales.

## Electronic supplementary material

Below is the link to the electronic supplementary material.

Supplementary material 1 (PDF 33 kb)
