# Supplementary material for: Legal Framework for Biosphere Reserves as Learning Sites for Sustainable Development: A Comparative Analysis of Ukraine and Sweden
Source: Ambio. 2013 Mar 10;42(2):174–87. doi: 10.1007/s13280-012-0373-3 (PMC3593038; doi:10.1007/s13280-012-0373-3)
Supplement: Supplementary file 1 — Supplementary material 1 (PDF 33 kb) [file 13280_2012_373_MOESM1_ESM.pdf]

Electronic Supplementary Material

Legal framework for biosphere reserves as learning sites for sustainable development: a comparative analysis of Ukraine and Sweden

Marine Elbakidze, Thomas Hahn, Volker Mauerhofer, Per Angelstam, Robert Axelsson

Table S1 Data on study areas in Ukraine and Sweden (source: Nomination form prepared by each of the 10 case study sites for submission to MAB National Committees in Ukraine and Sweden to become a BR)

	Ukraine					Sweden				
	Carpathian	Desnyanskyi	Eastern Carpathian (Uzhansky)	Roztochya	Western Polissya (Shatsky)	Blekinge Arkipelag	Kristianstad Vattenrike	Vänern archipelago with Kinnekulle	Nedre Dalälven River Landscape	East Vättern scarp landscape
Year of establishment	1991	2002	1999	2011	2002	2011	2005	2010	2011	Candidate
Total area:	53 630	70 748	39159	74416	48977	213 000 (156000 sea)	104 375	278 600	30 8750	105 520 (incl. 31800 – lake)
Core area	18 878 (35.2%)	2 397 (3.4%)	3 531 (9%)	3314 (4.5%)	5 145 (11%)	20962 (10%)	7179 (7%)	16281 (6%)	23400 (7.6%)	2120 (2%)
Buffer zone	15 928 (29.7%)	13 156 (18.6%)	5146 (13%)	10874 (14.5%)	12 971 (26%)	47212 (22%)	22900 (22%)	40876 (15%)	19350 (7%)	41800 (31800 – lake) (39%)
Transition zone	18 824 (35.1%)	55 195 (78%)	30 482 (78%)	60228 (81%)	30861 (63%)	144623 (67%)	74297 (71%)	221443 (79%)	266000 (86%)	61600 (59%)
Ecoregion	Mountain forests and alpine meadows	Temperate mixed forests and floodplains	Mountain forests	Temperate broad-leaf forests	Temperate mixed forests and floodplains	Temperate and sub-polar broadleaf forests or woodlands	Temperate and sub-polar broadleaf forests or woodlands	Temperate and sub-polar broadleaf forests or woodlands	Boreal coniferous forests	Deciduous broadleaf and coniferous forests
Number of red listed species	301 (national) 12 (EU BD) 202 (EU	39 (national) 11 (EU HD [illegible])	115 (national) 14 (EU HD)	154 (national) 156 (EU HD) 88 (EU BD	61 (national) 154 (EU HD)	499 (national) 13 (EU HD) 36 (EU	711 (national) 16 (EU HD) 43 (EU	171 (national) 18 (EU HD) 33 (EU	487 (national)	304 (national) 8 (EU HD) 38 (EU BD)

	HD)			□)		BD)	BD)	BD)		6 (global)
						18 (global)	22 (global)	20 (global)		
Main economic activities	Recreation Forestry Traditional farming	Agriculture Forestry Fishery Hunting Recreation	Forestry Agriculture Tourism Recreation	Agriculture Forestry Urban development	Agriculture Forestry Tourism Recreation Fishery	Forestry Agriculture Tourism Fishery	Agriculture Forestry Tourism	Agriculture Forestry Tourism	Forestry, forest industry Iron and steel industry Agriculture Tourism Fishing	Agriculture Forestry Hunting Fishing Tourism
Number of inhabitants:	100 000	2400	12 300	52377	14000	107085	68339	59561	65819	39690
Core area	0	0	0	0	0	256	14	7	36	47
Buffer zone	0	0	0	0	0	29657	1959	9280	4413	6945
Transition zone	100 000	2 400	12300	52377	14000	77172	66366	50274	61370	32698

□ EU HD (European Union Habitat Directive), EU BD (European Union Bird Directive)

Table S2 Selected key-words from the 'model law' (Bonnin and Jardin 2009) grouped to BRs' core functions

Core functions	Key-words	Total number
Conservation	'conservation', 'biosphere', 'ecologically sustainable', 'biological diversity', 'adaptive management', 'integrated management', 'natural heritage', 'long-term protection', 'ecological functions', 'ecological connectivity'	10
Development	'economic development', 'human development', 'socio-culturally sustainable', 'sustainable development', 'future generations', 'cultural heritage', 'multiple-use', 'sustainable use', 'ecologically healthy', 'economically viable', 'all concerned parties', 'private stakeholders, 'public stakeholders', 'social stakeholders', 'natural products', 'ecosystem services', 'adaptive management', 'integrated management', 'adaptive governance', 'appropriate technologies', 'traditional knowledge', 'local communities', 'participation', 'integrated management policy', 'integrated governance', 'coordinate', 'integrate', 'consultation', 'interaction', 'national and regional development policy', 'land development'	31
Logistic support	'exchange of experience', 'environmental education', 'local', 'regional', 'national', 'global', 'awareness', 'interdisciplinary', 'innovation', 'environmental monitoring', 'national communication'	11
